# *Nocardia farcinica* infection presenting as a solitary bronchial neoplasm in an immunocompetent adult: a case report

**DOI:** 10.3389/fmed.2023.1337303

**Published:** 2024-01-10

**Authors:** Yuying Tan, Mei Yang, Chun Wan, Shijie Tang, Lin Liu, Lei Chen

**Affiliations:** ^1^Department of Pulmonary and Critical Care Medicine, West China Hospital, Sichuan University, Chengdu, Sichuan, China; ^2^Department of Pulmonary and Critical Care Medicine, 363 Hospital, Chengdu, Sichuan, China

**Keywords:** bronchial neoplasm, metagenomic next-generation sequencing, *Nocardia farcinica*, postural dyspnea, immunocompetent adult

## Abstract

Nocardia species are gram-positive, acid-fast, saprophytic, aerobic bacilli, predominantly resulting in opportunistic infections in immunocompromised individuals. Here, we reported a case of Nocardia infection in a 27-year-old woman with normal immunocompetence, who presented as a solitary neoplasm in the left principal bronchus with a chief complaint of postural dyspnea. By electrotomy via bronchoscopy, the neoplasm was successfully removed, and it was further identified as *Nocardia farcinica* by metagenomic next-generation sequencing.

## Introduction

Nocardia species are gram-positive, acid-fast, saprophytic, aerobic bacilli, widely found in soil, decomposing vegetation, and other organic matter (1). Among all of the Nocardia species, *Nocardia asteroides*, *Nocardia brasiliensis*, and *Nocardia otitidiscaviarum* were documented to be the most common pathogenic strains (2), predominantly causing opportunistic infections in immunocompromised individuals (3). However, in the present report, we described a case of *Nocardia farcinica* infection in an immunocompetent adult, presenting as a solitary neoplasm in the left principal bronchus with a chief complaint of postural dyspnea.

## Case presentation

**Figure 1:**
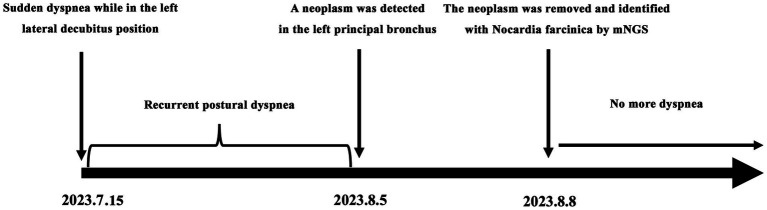
Timeline of the patient’s history of present illness.

**Figure 2:**
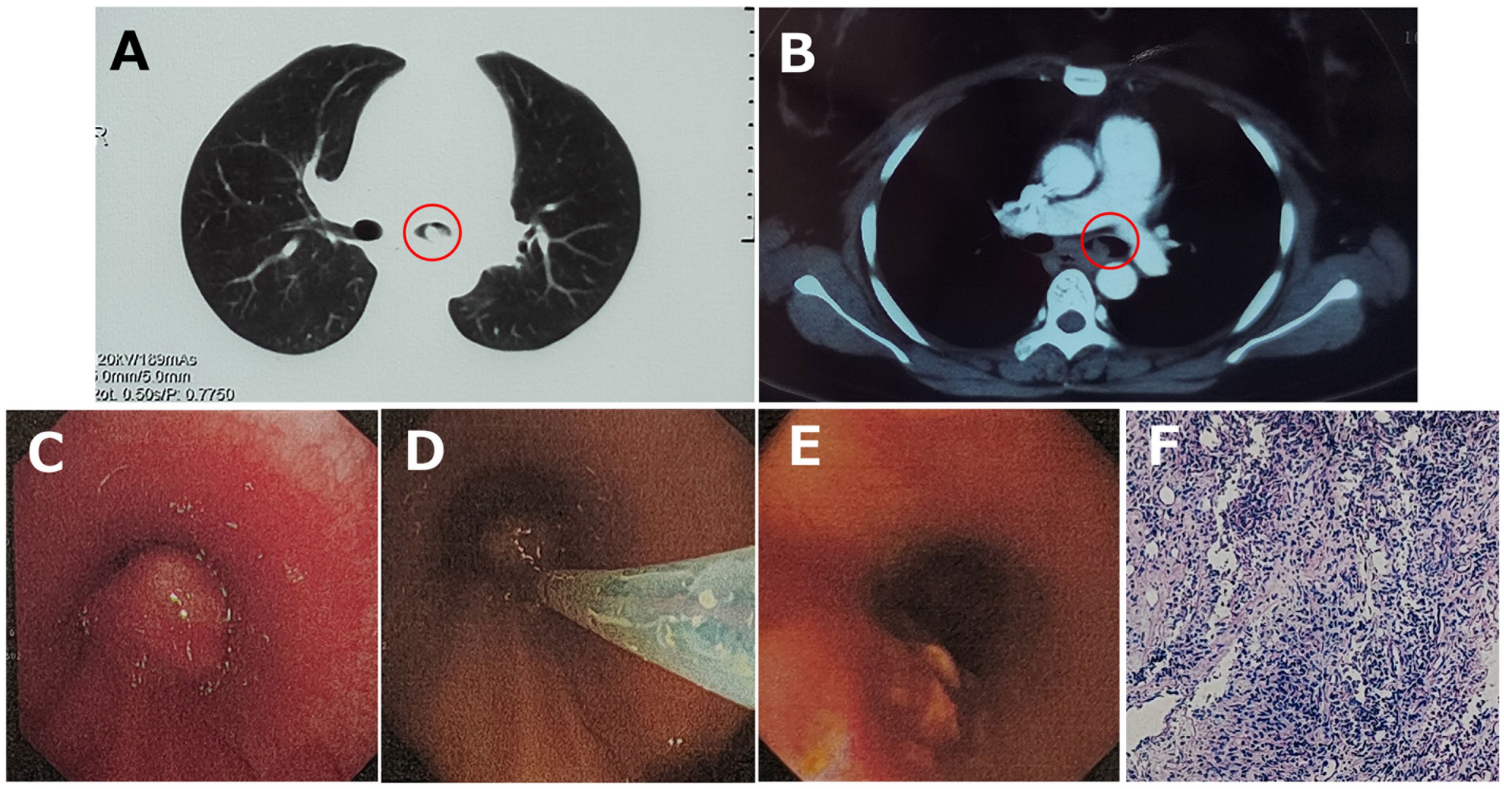
**(A)** Chest tomography (CT) displayed a small hyperdense nodule (red circles) in the left principal bronchus, and **(B)** mild intensification was detected in the enhanced CT image. **(C)** Bronchoscopy visualized a smooth-surfaced neoplasm with airway obstruction in the left principal bronchus, and **(D)** it was excised with electrotomy and **(E)** removed successfully. **(F)** HE stain showed chronic active inflammation with histiocyte aggregation.

A 27-year-old woman was admitted to the local hospital because of recurrent dyspnea while in the left lateral decubitus position for 3 weeks. No other symptoms such as fever, cough, wheezing, expectoration, hemoptysis, and chest pain were reported. Moreover, she had a history of surgery in the right talus due to cartilage damage 5 months ago and denied any history of asthma, bronchiectasis, pulmonary tuberculosis, allergic diseases, anemia, autoimmune diseases, acquired immune deficiency syndrome, and tumors. On physical examination, no positive signs were revealed. The timeline of history for the present illness was showcased in Figure 1. In laboratory tests, the interferon-gamma release assay was positive, but the tuberculin skin test was negative. Furthermore, there were no positive results in the blood routine examination, antinuclear and anti-neutrophil cytoplasmic antibody test, and human immunodeficiency virus antibody test (Table 1). Importantly, chest tomography (CT) displayed a small hyperdense nodule of approximately 5 mm × 6 mm in the left principal bronchus with mild intensification in the enhanced images (Figures 2A,B). No significant lesions were detected in the mediastina, lung lobes, and pleural cavities.

**Table 1:** Results of laboratory tests.

Laboratory tests	Results	Reference values	Units
WBC	9.25	3.5–9.5	10*9/L
RBC	5.08	3.8–5.1	10*12/L
HGB	141	115–150	g/L
HCT	41.4	35–45	%
MCV	81.5	82–100	fL
MCH	27.8	27–34	pg
MCHC	341	316–354	g/L
RDW	13.1	11.4–14.5	%
PLT	286	125–350	10*9/L
NEUT	6.11	1.8–6.3	10*9/L
LYMP	2.45	1.1–3.2	10*9/L
MON	0.54	0.1–0.6	10*9/L
EOS	0.11	0.02–0.52	10*9/L
BASO	0.04	0–0.06	10*9/L
NRBC	0	0–0.03	10*9/L
PT	9.4	10-13	sec
INR	0.81	0.8–1.3	
APTT	22.5	23–40	sec
FIB	2.4	2–4	g/L
TT	18.3	14–21	sec
FDP	1.6	0–5	ug/mL
D-Dimer	0.36	0–1	mg/L
ANA	(−)	(−)	
ANCA	(−)	(−)	
HIV-Ag/Ab	0.27	<1	COI
IGRA	(+)	(−)	
TST	(−)	(−)	

ANA, antinuclear antibody; ANCA, anti-neutrophil cytoplasmic antibody; APTT, activated partial thromboplastin time; BASO, basophils; EOS, eosinophils; FDP, fibrin degradation product; FIB, fibrinogen; HCT, hematocrit; HGB, hemoglobin; HIV-Ag/Ab, human immunodeficiency virus antigen/antibody; IGRA, interferon-gamma release assay; INR, international normalized ratio; LYMP, lymphocyte; MCH, mean corpuscular hemoglobin; MCHC, mean corpuscular hemoglobin concentration; MCV, mean corpuscular volume; MON, monocyte; NEUT, neutrophil; NRBC, nucleated red blood cell; PLT, platelet; PT, prothrombin time; RBC, red blood cell; RDW, red blood cell distribution width; TST, tuberculin skin test; TT, thrombin time; WBC, white blood cell.

By endotracheal endoscopy, a smooth-surfaced neoplasm with airway obstruction was visualized in the left principal bronchus (Figure 2C). Subsequently, the neoplasm was successfully removed with electrotomy (Figures 2D,E), and pathological biopsy with metagenomic next-generation sequencing (mNGS) was then carried out for precise diagnosis.

The hematoxylin-eosin (HE) staining showed chronic active inflammation, evidenced by granulation tissue hyperplasia, inflammatory necrosis, and exudation, as well as histiocyte aggregation with a tendency of granuloma formation (Figure 2F). mNGS was conducted by Beijing Genomics Institute (BGI), Shenzhen. First, total DNA from the tissue sample was isolated, and after DNA library construction, sequencing was performed by the MGISEQ-2000 platform (BGI) (4, 5). Then, short reads, duplicates, and human host sequences were removed using the Burrows-Wheeler Alignment Tool. Finally, the remaining sequences were compared with the PMseq metagenomic database (PMDB, BGI), consisting of bacteria, fungi, viruses, and parasites, which indicated the significant presence of *Nocardia farcinica* (14,850 reads), with possible contamination or colonization by *Pseudomonas stutzeri* (22 reads), *Cutibacterium acnes* (61 reads), *Kocuria palustris* (34 reads), *Acinetobacter ursingii* (14 reads), and *Staphylococcus hominis* (3 reads; Table 2).

**Table 2:** Results of metagenomic next-generation sequencing.

Type	Generic name	Standardized reads	Specific name	Standardized reads	Relative abundance
G+	Nocardia	19,231	*Nocardia farcinica*	14,850	13.73%
G−	Pseudomonas	111	*Pseudomonas stutzeri*	22	0.13%
G+	Cutibacterium	76	*Cutibacterium acnes*	61	0.11%
G+	Kocuria	39	*Kocuria palustris*	34	0.07%
G−	Acinetobacter	27	*Acinetobacter ursingii*	14	0.02%
G+	Staphylococcus	27	*Staphylococcus hominis*	3	0.01%

G+, Gram-positive; G−, Gram-negative.

Overall, the patient was definitely diagnosed with *Nocardia farcinica* infection localized in the left principal bronchus. Moreover, since no clinical evidence supported the possibilities, tuberculosis and tumors were excluded. After removal of the neoplasm and during the follow-up, the patient had no more dyspnea. Therefore, no antibiotic therapy was administrated all the way.

This case report was approved by the Institutional Review Board of West China Hospital of Sichuan University, and the informed consent was obtained.

## Discussion

Nocardia species are highly opportunistic pathogens and typically cause infections in immunocompromised individuals (6–9). Unlike the other strains, *Nocardia farcinica* often exhibits pulmonary infection via inhalation with a greater propensity for dissemination and a unique pattern of antibiotic susceptibility (10). Consequently, it is prone to develop into severe pneumonia or sepsis, leading to a deteriorating prognosis (11, 12).

However, it has been reported that pulmonary infection by *Nocardia farcinica* may occur in immunocompetent subjects (Table 3) (13–16). Although these reported cases typically presented with fever, cough, and expectoration, as well as leukocytosis and elevated C-reactive protein (CRP), radiological findings usually demonstrated the localized presence of nodular shadows and pleural effusion, which was more easily recovered after antibiotic treatment. Differently, this patient, an immunocompetent host, complained of postural dyspnea but no fever and other respiratory symptoms, and the *Nocardia farcinica* infection was strictly limited within the left principal bronchus, presenting as a solitary neoplasm.

**Table 3:** Clinical characteristics of pulmonary infection by *Nocardia farcinica* in immunocompetent cases.

Study	Country	Gender	Age	Symptoms	Lab tests	Chest CT	Identification	Antibiotics	Outcome
Babayigit et al. (13)	Turkey	Female	13	Fever/Cough/Expectoration/Hemoptysis	Leukocytosis/Elevated CRP	Multilobar nodular lesions	Sputum culture	TMP–SMZ/MEM	Recovery
Kim et al. (14)	Korea	Male	64	Chest pain/Dyspnea	Leukocytosis/Elevated CRP	Bilateral pleural effusion/A mass in the right pericardium	16S rRNA sequencing	TMP–SMX/PEM	Recovery
Bai et al. (15)	China	Male	55	Fever/Cough/Expectoration/Chest pain	Not reported	Patches in bilateral upper lobes	16S rRNA sequencing	TMP–SMX	Recovery
Dong et al. (16)	China	Male	59	Fever/Cough/Expectoration	Leukocytosis/Elevated CRP	Multiple patches and nodules in both lungs	mNGS	SMZ	Recovery
Present case	China	Female	27	Postural dyspnea	No leukocytosis	A nodule in the left principal bronchus	mNGS	Not used	Recovery

AMK, amikacin; CRP, C-reactive protein; CT, chest tomography; MEM, meropenem; mNGS, metagenomic next-generation sequencing; PEM, prepenem; SMZ, sulfamethoxazole; TMP–SMZ, trimethoprim–sulfamethoxazole.

The precise identification of Nocardia species has been challenging. In the past decades, traditional methods including microscopic examination, microbial culture, and biochemical analyses were widely used, but with more false-negative results than expected (17, 18). Subsequently, 16S rRNA sequencing seems to improve the testing efficiency; however, this technique might miss the potential non-bacterial infections that are usually considered depending on the clinical assumption (19, 20). More importantly, the clinical presentation of this patient was so atypic that we could not exclude the possibility of non-bacterial infections. Thus, for this case, 16S rRNA sequencing may not be the best choice for microorganism identification, and relatively, mNGS is a more ideal method that, regardless of clinical assumption, identifies pathogenic microorganisms/Nocardia strains comprehensively, quickly, and accurately, providing an opportunity for precise interventions at the early stage (21–23). As demonstrated in this case, the significant presence of *Nocardia farcinica* was detected by mNGS from the resected tissue, which confirmed its pathogenic role according to the Johns Hopkins ABX Guide (24). However, it should be noted that mNGS reports must be cautiously interpreted before accurate differentiation of pathogenic, contaminated, and colonized subgroups (19).

Moreover, antimicrobial therapy plays a key role in the treatment of Nocardia infections. Trimethoprim–sulfamethoxazole (TMP–SMX) is the most recommended antibiotic, usually combined with imipenem and amikacin (25). However, as suggested in this case, antibiotics are not always necessary to treat the localized infections, but surgical resection can bring about a significant improvement and recovery.

## Conclusion

Overall, this case report indicates (i) immunocompetent individuals are the target for pulmonary infection by Nocardia, which may present as atypical manifestations and localized lesions; (ii) adoption of mNGS can benefit for accurate identification and early diagnosis of Nocardia infections; and (iii) surgical management but not antibiotic therapy could be the first choice for these patients with localized lesions.

## Data availability statement

The original contributions presented in the study are included in the article/supplementary material, further inquiries can be directed to the corresponding authors.

## Ethics statement

Written informed consent was obtained from the individual(s) for the publication of any potentially identifiable images or data included in this article.

## Author contributions

YT: Writing – original draft, Data curation, Software, Visualization. MY: Writing – original draft, Data curation, Visualization. CW: Writing – original draft, Data curation, Formal Analysis. ST: Formal Analysis, Writing – original draft. LL: Writing – review & editing, Conceptualization. LC: Writing – review & editing, Conceptualization, Supervision, Writing – original draft.
